# The mediating effect of personality on mental workload and perceived professional benefits of nurses in East China

**DOI:** 10.1186/s12912-023-01603-3

**Published:** 2023-11-22

**Authors:** Ling Li, Zhixian Feng, Mingling Zhu, Jialu Yang, Lili Yang

**Affiliations:** 1 School of nursing, Zhejiang Shuren University, 8 Shuren Road, 310015 Hangzhou, ZheJiang PR China; 2 School of nursing, Zhejiang Chinese Medical University, 548 Bin-wen Road, 310053 Hangzhou, Zhejiang PR China

**Keywords:** Nurse, Mental workload, Personality, Perceived professional benefits, Cross-sectional survey

## Abstract

**Background:**

Nursing work is a work with high-stress load, and nurses with different personality may have different subjective feelings about their workload. Therefore, it is particularly necessary to comprehend the perceived professional benefits of nurses engaged in nursing work under high-pressure background, especially during the epidemic period. This study explored the relationship between mental workload, personality, and perceived professional benefits of nurses, and offer advices for the intervention of nurses with different personality to improve their perceived professional benefits.

**Materials and methods:**

In this study, we used a cross-sectional study with a convenient sampling. 473 in-service nurses in Class A tertiary hospitals of Zhejiang Province were recruited by using the NASA Mission Load Index scale of nurses, the brief version of China’s Big Five Personality Questionnaire, and the Nurses’ perceived professional benefits questionnaire from July 2020 to March 2021. Sample size is 54.91%, and the response rate is 100%. Cronbach’s alpha method was used to evaluate the reliability of the instruments. Descriptive statistical analysis was used to describe the socio-demographic data of the subject, and scores for research variables. The Mann-Whitney U-test, and Kruskal-Wallis H rank-sum test were used to compare the scores of perceived professional benefits with different demographic characteristics. Correlation analysis results were presented as the Spearman correlation coefficient. The plug-in v2.16.3 provided by SPSS software was used for linear regression analysis, and the deviation-corrected percentile Bootstrap method was used to examine the mediating role of personality (neuroticism, conscientiousness, agreeableness, openness and extroversion).

**Results:**

Age, length of service in nursing, and record of formal schooling can affect nurses’ perceived professional benefits. Mental workload, and perceived professional benefits were all above the median value. The mental workload was negatively correlated with perceived professional benefits (r= -0.129, P < 0.01), positively correlated with neuroticism (r = 0.242, P < 0.01), negatively correlated with agreeableness, openness, extroversion (r=-0.229~-0.221, P < 0.01), and negatively correlated with conscientiousness, but the differences were not significant. Nurses’ perceived professional benefits were negatively correlated neuroticism (r=-0.109, P < 0.05), but positively associated with conscientiousness, agreeableness, openness, and extroversion (r = 0.098 ~ 0.326, P < 0.05). The mental workload can directly affect the perceived professional benefits in the direct effects, and can also affect the it through the mediating effect of agreeableness, extroversion, neuroticism, and openness.

**Conclusions:**

Age, length of service in nursing, and record of formal schooling could affect nurses’ perceived professional benefits, and personality played a partial mediating role in the influence of mental workload on the perceived professional benefits. The results of this study can provide strategies for nurses’ human resource management. According to different demographic factors, and personality, various measures should be taken to guide nurses to evaluate the mental workload correctly, reduce their emotional pressure, increase job resources, and improve their perceived professional benefits.

## Introduction

On January 26, 2020, the National Health Committee issued the new coronavirus infection of pneumonia pandemic emergency psychological crisis intervention guidelines stressing the importance of coping with psychological crises during the COVID-19 pandemic [1]. With the normalization of the COVID-19 epidemic, the National Health Commission of China (NHCC) released the principles of Psychological Crisis Intervention in Emergency Response to SARS-CoV-2 Pneumonia emphasized that the priority for possible psychological problems during epidemic prevention should start from the first level of the population, nurses who work on the clinical front line [2], who completed routine nursing work, should also undertake epidemic prevention tasks in addition. Such as nurses are not only participating in health services providing nursing care to patients with severe COVID-19 tirelessly on the front line, but also participating in COVID-19, and health education campaigns to verify compliance with measures to prevent transmission [3]. Due to the particularity of their posts, the work intensity, risk, and pressure of this group were all higher than other posts during the epidemic [4]. Such as, long-term separation from family members, heavier protective measures, higher risk of infection, lack of protective materials, and greater psychological pressure, etc. [5].

Workload is generally considered to be the ratio of requirements to available resources. It includes objective workload, and subjective workload, which in turn refers to the subjective psychological experience of human operators when performing tasks under specific environmental or operating conditions, also known as mental workload [6]. Mental workload refers to the amount of psychological effort required by workers to complete a task, involving many factors such as subjective process, task demand, external support, worker cognition, and past experience [7, 8]. Appropriate mental workload is an important guarantee for medical staff to make correct clinical decisions, and ensure patient safety [9]. However, excessive mental workload will affect physical skills, and lead to fatigue, and functional errors, resulting in changes in behavior, and work performance [8].

Nurses’ perceived professional benefits (PPB) refers to the positive emotional state. Based on positive psychology’s theory, the nurses feel satisfied with the harvest, and benefits brought by their profession in the process of practice it could promote their overall growth, and agree with the nursing profession [10]. The stronger the sense of benefit, the more love nurses have for nursing work. When the nurses got a positive experience, met the professional expectation, and improved their sense of professional benefits in the clinical nursing experience, the turnover rate can be effectively reduced, they can improve their professional identity as nursing staff, and be more engaged in nursing work, provide patients with higher quality care [11, 12]. And studies have confirmed a correlation between PPB, and well-being [10]. Job demand-resource model (JD-R model) think that job conditions are divided into two parts, job demands, and job resources. The former refers to physical, psychological, social, or organizational aspects of the job that require effort and /or skill; The latter refers to the physical, mental, social or organizational, and other aspects of work, which helps to stimulate inner motivation, achieve goals, and reduce work stress [13]. Both of these job characteristics may influence employee distress levels, motivation, job engagement, and job satisfaction [14, 15]. The mental workload can be regarded as a job demand, and the PPB is a kind of job resource. Previous studies have studied them independently, and whether these two job conditions affect each other through other factors is unclear.

As a person’s dynamic, internal psychological organization, personality can create a person’s thoughts, feelings, actions, and other characteristic patterns [16]. Personality is a dynamic organizational structure that can determine a person’s behavior, and way of thinking in the psychological system [17]. It can not only affect an individual’s interpersonal relationship, behavior style, and social adaptation, but also is an important indicator to predict an individual’s career achievement [18]. Costa proposed a five-factor model containing neuroticism, extraversion, openness, agreeableness, and conscientiousness [19]. The perception of stress depends not only on the stressor itself, but also on the personality, and coping style of the individual when faced with a stressful situation, especially subjective stress, which has been recognized as a vital component of maladaptive personality traits [20, 21]. Due to better adaptive coping, social support, and positive emotions, individuals with agreeableness, extroversion, conscientiousness, and openness are more likely to obtain higher levels of resilience [22], and thus gain better professional benefits in the work environment. A review of previous studies showed that (1) mental workload is correlated with PPB. Zeng’s research outcome showed that PPB is negatively correlated with pressure load, and mental health [23]. (2) Some correlations exist between personality, and PPB. Li’s research found that the PPB, and self-efficacy scores of practice nurses were significantly correlated with psychoticism, introversion, extroversion, emotional stability, and lie scale [18]. The top five personality of nurses are predictors of job burnout, especially neuroticism is significantly negatively correlated with job engagement [24, 25]. A study of obstetrics, and gynecology nurses found that positive personality, such as extroversion, and openness improved the nurses’ empathy satisfaction, and neuroticism, and agreeableness were influential factors for empathy fatigue [26]. (3) Mental workload is related to personality. The mental workload can be a mediator which is influenced by personality [27]. Zhan held that there was a significant negative correlation between workload, and happiness level of Guangdong railway police, and personality played an intermediary role, and there was a significant positive correlation between workload, psychoticism, and neurotic factors in personality [28]. (4) The personality played a partial mediating effect between perceived social support, and subjective well-being [29], and personality, and mental health play a chain mediating role between cognitive ability, and English learning performance [30]. Personality had a mediating role between childhood abuse, and depressive symptoms [31]. Based on previous studies, we believe that nurses with different personality should be helped to find ways to obtain, and retain resources constantly when coping with work pressure, reduce the perception of workload, and experience the PPB of medical staff in the era of the epidemic, thus increasing job engagement. Through which kind of personality does mental workload affect PPB? What is the extent of its impact? It is not clear which aspects nursing managers should focus on adjusting mental workload according to different personalities to improve their perceived professional benefits. There are few studies on the nurses’ PPB during the pandemic, especially the influence of nurses with different personalities on PPB in the face of mental workload.

## Methods

### Aim & hypotheses

The study aimed to investigate the status quo of mental workload, personality, and PPB of nurses in tertiary hospitals, and the influencing factors of social-demographic characteristics on PPB, and to explore the mediating role of personality in mental workload, and PPB.

Therefore, the researchers of this study proposed a hypothesis.

**H1**

Mental workload is correlated with perceived professional benefits of nurses.

**H2**

Mental workload has a relationship to personality (neuroticism, extraversion, openness, agreeableness, and conscientiousness) of nurses.

**H3**

Personality (neuroticism, extraversion, openness, agreeableness, and conscientiousness) is related to perceived professional benefits of nurses.

**H4**

Personality (neuroticism, extraversion, openness, agreeableness, and conscientiousness) mediate the relationship between mental workload, and perceived professional benefits of nurses.

**Fig. 1:**
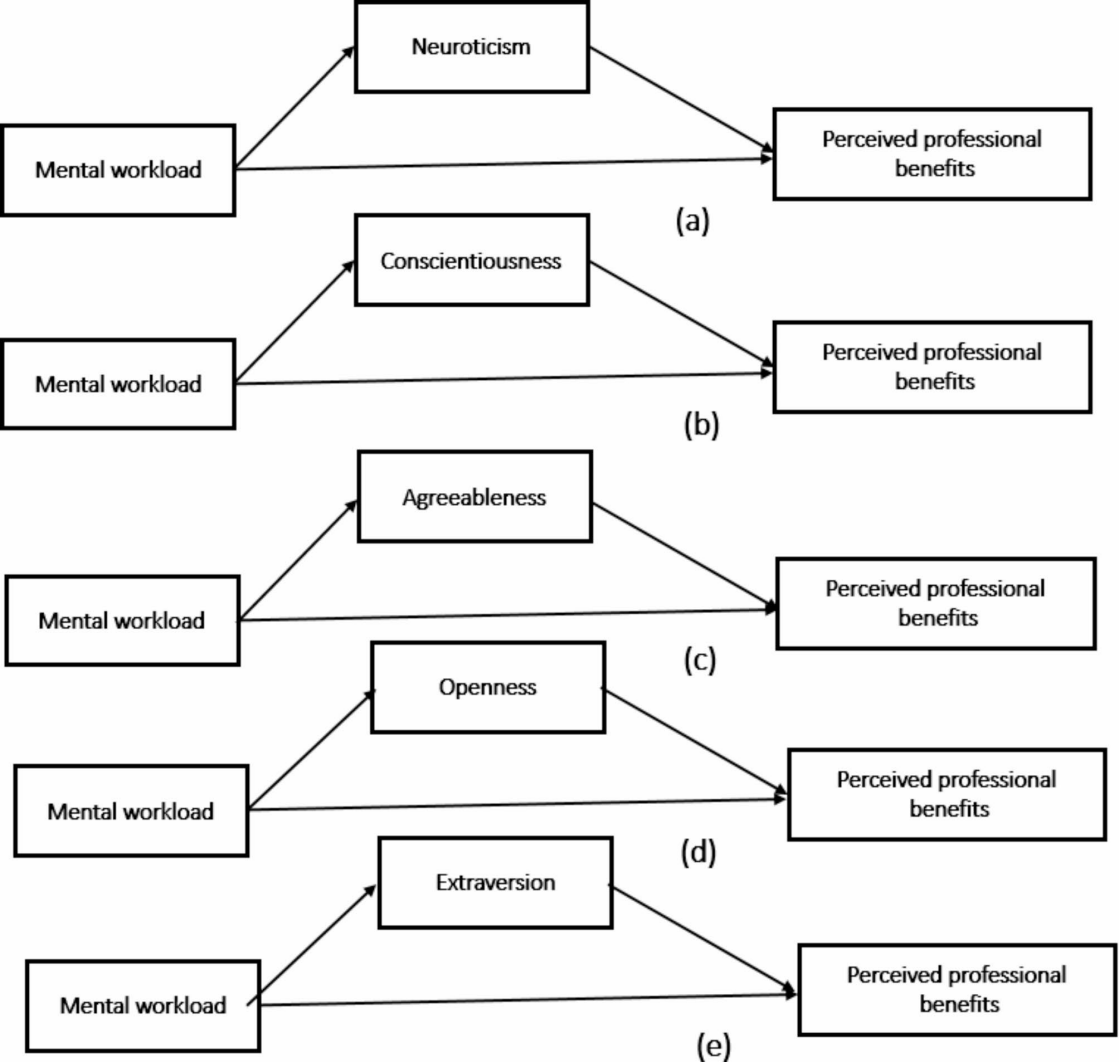
Research theoretical hypothesis diagram

The theoretical hypothesis proposed in this study is shown in Fig. 1.

### Study design

We conducted a cross-sectional design adopting a convenient sampling method in compliance with the Strengthening the Reporting of Observational Studies in Epidemiology (STROBE) guidelines [32]. An anonymous online self-report survey in Chinese was conducted between July 2020, and March 2021 using the Questionnaire star survey platform, which is a free online questionnaire survey platform. The online website of this survey is: https://www.wjx.cn/wjx/design/previewmobile.aspx?activity=98877264&s=1.

### Sample and sampling method

The formula N=(Z_1−α/2_ × δ/ σ)^2^ for calculating the sample size of measurement data in a cross-sectional survey is based on the following assumptions: the PPB of the specialist nurses in a tertiary hospital is 127.36 ± 23.65 [33], and the tolerance error is 5, then N=(1.96 × 23.65/5)^2^= 85.947 ≈ 86. The non-response rate was increased by 20%, and the final sample size of 115 nurses was 86 × (1 + 0.2)/0.9, assuming a questionnaire pass rate of 90%. Sampling was conducted by convenience sampling; participants were all registered nurses who were working in 12 Class A tertiary hospitals in Hangzhou. An explanation for the selection, and inclusion of study samples are provided in Additional file 1. The inclusion criteria were nurses who had obtained registered nurse qualification certificate; Nurses engaged in clinical nursing (including clinical nursing, and nursing management); Nurses who agreed to participate in the study. Exclusion criteria included nurses who were not primarily involved in clinical care, and nurses who were unable to participate in the study due to leaving, or studying abroad during the survey period.

### Data collection instruments

The demographic characteristics questionnaire was designed by the researchers, including working hospital, gender, age, labor and personnel relations, length of service in nursing, working department, record of formal schooling, professional title, marital status.

NASA Mission Load Index Scale for Nurses [34]: The National Aeronautics and Space Administration Task Load Index (NASA-TLX) developed by Hart [35] is a subjective evaluation scale of psychological load, which is mostly used in human ergonomics studies. The Chinese version of the NASA-TLX scale, adapted by Liang [34] has 6 items: mental demand, physical demand, temporal demand, performance, effort, frustration, which forms two dimensions, load feeling, and self-assessment. Each entry is represented by a line of 20 equal points, representing 0 to 100 points respectively, and a line from 0 to 100 indicates a load from “low” to “high” respectively. The item “performance” is represented by “perfect” to “failure” from left to right, that is, the lower the score, the more perfect the self-expression, the lower the task load; The higher the score, the more self-performance failure, the higher the task load. The dimension is divided into the arithmetic mean of the corresponding item score, and the total score is the arithmetic mean of all the item score. The scale has good reliability, and validity (retest reliability was 0.806, The Cronbach’s alpha coefficient was 0.707, and internal consistency coefficient was 0.782 [34]). The Cronbach’s alpha coefficient of the scale was 0.756 in this study.

Nurses’ perceived professional benefits Questionnaire [36]. The questionnaire was compiled by Hu Jing [37], with a total of 33 items, and 5 dimensions, including positive professional perception, good patient-nurse relationship, recognition from families, and friends, a sense of belonging to the work team, and personal growth. Five-point Likert scale was used for accessing attitude, and practice scores, “strongly disagree”, “disagree”, “uncertain”, “more agree”, and “strongly agree” correspond to 1 ~ 5 points, and the score ranges from 33 to 165 points, with a total of 33 items. The content validity of the questionnaire ranged from 0.83 to 1.00, Cronbach’s α coefficients of each dimension ranged from 0.821 to 0.893, and the overall Cronbach’s α coefficient was 0.958. The Cronbach’s α coefficient was 0.840, and the Cronbach’s α coefficient of each dimension was 0.820 ~ 0.828. The Cronbach’s α coefficient of the scale was 0.917 in this study.

The Chinese Big Five Personality Inventory Brief Version (CBF-PI-B). The questionnaire was compiled by Wang et al. [38] on the basis of the Chinese Big Five Personality Questionnaire (CBF-PI), which is a self-rating scale, consisting of five dimensions, Neuroticism (N), Extraversion (E), Openness (O), Agreeableness (A), and Conscientiousness (C). It consists of 40 items using a five-point Likert scale. Six points scoring method was adopted, including 7 reverse-scoring questions. Cronbach’s α coefficients of the five dimensions were all above 0.75, the minimum dimension (agreeableness) was 0.76, the maximum dimension (neuroticism) was 0.81, and the mean value of each dimension was 0.79. Retest coefficients showed a minimum of 0.67 for agreeableness, a maximum of 0.81 for neuroticism, and a mean of 0.74 for the five dimensions after a 10-week interval. The Cronbach’s α coefficient of the scale in this study was 0.797.

### Data collection method

This study has been approved by the Ethics Committee of the University, then the researchers sent the link of the questionnaire to the nurses who were willing to participate in the survey through the nurses working in the hospitals. All participants gave informed consent, and completed the questionnaire independently. Their names, and identities were not revealed in this study.

### Ethical considerations

This study protocol was approved by the Institutional Review Board and Ethics Review Committee of Zhejiang Shu Ren University (NO. 20,200,608). The research carried out in accordance with the Declaration of Helsinki and its subsequent amendments. Informed consent was obtained from all subjects, and the nurses were assured that participation was voluntary, and that they could withdraw at any time, without penalty, if they wished.

### Data analysis

IBM SSPS 25.0 software was used to analyse the data in this study, descriptive statistical analysis was first used to consider the characteristics of samples, and variables using. Then, the normality test results showed that the normal distribution was unsatisfactory. Measurements that meet the normal distribution are described in xj±$\pm$s, while those that do not meet the normal distribution are described in the distance between median, and quartile. The Mann-Whitney U-test, and Kruskal-Wallis H rank-sum test were used to compare the scores of PPB with different demographic characteristics. Spearman correlation analysis was used to illustrate the correlation of various variables. Linear regression analysis in plug-in process v2.16.3 provided by IBM SSPS 25.0 software, and deviation-corrected percentile Bootstrap method were used to examine the mediating effect of personality (neuroticism, conscientiousness, agreeableness, openness, and extroversion)on mental workload, and PPB with 95% confidence interval based on 5000 samples.The p-values less than 0.05 were considered statistically significant.

## Results

Most respondents were female (n = 425, 89.85%). Nurses aged between 25 and 35years old accounted for the largest proportion(n = 244,51.59%). Those whose labor and personnel relations were having establishment (In China, public hospitals belong to the establishment of public institutions, and nurses are divided into two types: with, and without establishment. The establishment of public hospitals is equivalent to the employees recognized by the state, reported, and registered, with good welfare benefits, retirement remuneration, and low risk of unemployment. Once they lose their jobs due to the dissolution of hospitals or other reasons, the state needs to arrange other jobs for them. On the other hand, the employee without establishment signs a labor contract with the hospital, and the welfare benefits are lower than that of the employees with establishment, the risk of unemployment is higher, and the pension is lower than those employees with establishment) were the majority (n = 304, 64.27%). The number of years of nursing practice is 2–5 years, 6–10 years, 11–20 years, accounting for almost the same proportion. A larger proportion of nurses worked in internal medicine, and surgery, 30.23%, and 26.64%, respectively. Nurses with undergraduate records of formal schooling occupied the mainstream (n = 415, 87.74%). In terms of job title, most nurses were nurse-in-charge (205, 43.43%). A higher proportion were married (n = 283, 59.83%). The demographic characteristics of participants, and the comparison of PPB scores between different subgroups are shown in Table 1. (Table 1).

**Table 1:** The demographic characteristics of participants, and the comparison of perceived professional benefits scores between different subgroups (N = 473)

Variables	N (%)	PPB score M(P25,P75)	H(K)Z value	Pvalue	Post hoc Test	
Gender						
Male	48(10.15)	128.50(110.25,143.00)	0.17	0.86		
Female	425(89.85)	128.00(116.00,145.00)				
Age						
<25 years old	84(17.76)	125.50(114.25,140.00)	9.79	0.02	2>4	
25-35years old	244(51.59)	129.00(115.00,141.00)				
36-40years old	89(18.82)	128.00(122.00,134.00)				
>40 years old	56(11.84)	120.00(105.00,133.75)				
Labor and personnel relations						
Have establishment	304(64.27)	129.00(116.00,140.00)	-1.94	0.05		
Without establishment	169(35.73)	122.00(114.00,140.00)				
Length of service in nursing						
<2 years	77(16.28)	131.00(115.00,143.00)	12.29	0.02	1,3>5; 3>4; 2<3”	
2–5 years	114(24.10)	127.00(115.00,136.50)				
6–10 years	120(25.37)	130.00(116.00,142.00)				
11–20 years	112(23.68)	128.00(113.00,134.00)				
>20 years	50(10.57)	126.00(107.75,133.00)				
Working department						
Internal medicine	143(30.23)	126.00(116.00,136.00)	6.27	0.39		
Surgery	126(26.64)	128.00(115.50,139.00)				
Outpatient service	19(4.02)	128.00(107.00,134.00)				
Emergency department	11(2.33)	140.00(108.00,143.00)				
ICU	43(9.09)	126.00(114.00,134.00)				
Pediatrics	17(3.59)	120.00(115.00,148.00)				
Other	114(24.10)	129.00(115.00,141.50)				
Record of formal schooling						
Technical secondary school	11(2.33)	134.00(119.00,159.00)	11.45	0.01	1,3>2	
Specialized subject	20(4.23)	119.00(114.00,126.00)				
Undergraduates	415(87.74)	128.00(115.00,140.00)				
Master degree or above	27(5.71)	120.00(112.00,130.00)				
Professional title						
The nurse	75(15.86)	126.00(116.00,140.00)	0.29	0.96		
Nurse practitioner	176(37.21)	127.00(113.00,141.00)				
Nurse-in-charge	205(43.34)	128.00(116.00,138.00)				
Deputy director nurse or above	17(3.59)	127.00(114.50,135.00)				
Marital status						
Unmarried	164(34.67)	128.00(118.00,140.00)	5.32	0.26		
Married	283(59.83)	127.00(113.00,139.00)				
Divorced	9(1.90)	122.00(109.00,128.00)				
Death of a spouse	6(1.27)	134.00(102.00,149.00)				
Remarried	11(2.33)	129.00(129.00,146.00)				

The median value of mental workload, and PPB are 78.00, and 128.00. The median values of neuroticism, conscientiousness, agreeableness, openness, and extroversion are 26.00, 32.00, 31.00, 31.00, and 29.00, respectively. The results of descriptive statistical analysis of variable scores are shown in (Table 2).

**Table 2:** Descriptive statistical analysis of variable scores

Variables	Scoring range	Total score, median(P25, P75)
Mental workload	0~120	78.00(71.00,86.00)
Perceived professional benefits	33~165	128.00(115.00,140.00)
Neuroticism	8~48	26.00(21.00,29.00)
Conscientiousness	8~48	32.00(29.00,35.00)
Agreeableness	8~48	31.00(29.00,33.00)
Openness	8~48	31.00(28.00,33.00)
Extroversion	8~48	29.00(27.00,32.00)

The mental workload of nurses was negatively correlated with the PPB (r= -0.129, P < 0.01), the mental workload was positively correlated with neuroticism (r = 0.242, P < 0.01), and negatively correlated with agreeableness, openness, extroversion (r=-0.229~ -0.221, P < 0.01), and negatively correlated with conscientiousness (r=-0.044), but the difference was not significant. PPB was negatively correlated with neuroticism (r=-0.109, P < 0.05), but positively correlated with conscientiousness, agreeableness, openness, and extroversion (r = 0.098 ~ 0.326, P < 0.05). See (Table 3) for details.

**Table 3:** Correlation analysis of research variables

	1	2	3	4	5	6	7
1	1						
2	-0.129^**^	1					
3	0.242^**^	-0.109^*^	1				
4	-0.044	0.319^**^	-0.148^**^	1			
5	-0.229^**^	0.326^**^	-0.209^**^	0.478^**^	1		
6	-0.221^**^	0.127^**^	-0.182^**^	0.221^**^	0.331^**^	1	
7	-0.229^**^	0.098^*^	-0.044	0.257^**^	0.378^**^	0.711^**^	1

Note: 1 to 7 are mental workload, perceived professional benefits, neuroticism, conscientiousness, agreeableness, openness, and extroversion, respectively

**Fig. 2:**
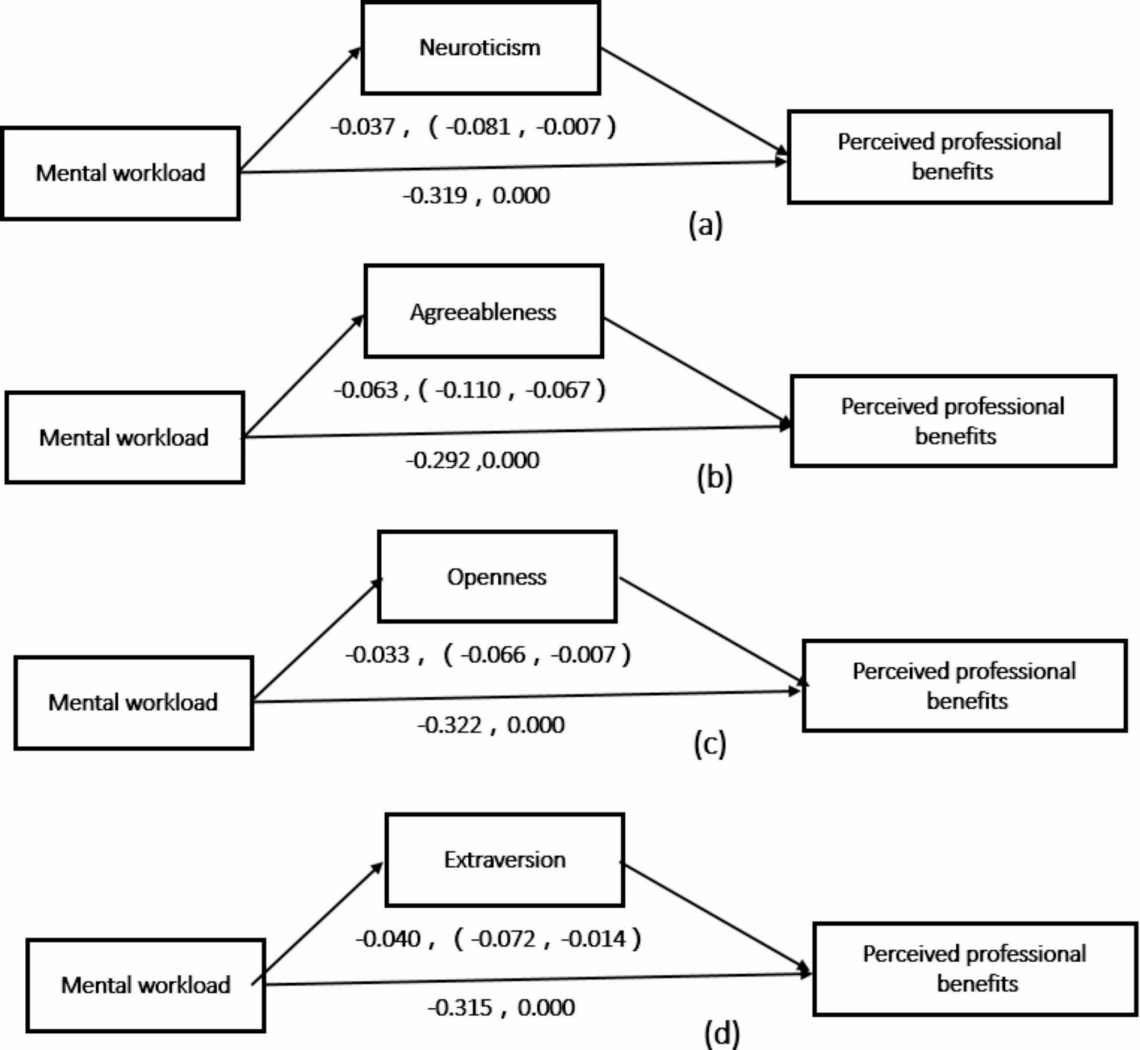
Path analysis models of mental workload, and personality on perceived professional benefits of nurses, (**a**), (**b**), (**c**), (**d**) is the mediating effect of neuroticism, agreeableness, openness, and extraversion in personality

After controlling for the variables gender, age, labor and personnel relations, length of service in nursing, working department, record of formal schooling, professional title, and marital status, we take mental workload as the independent variable, PPB as the dependent variable, and personality (neuroticism, conscientiousness, agreeableness, openness, and extroversion) as the mediating variables, Linear regression analysis in plug-in process v2.16.3 provided by IBM SSPS 25.0 software, and deviation-corrected percentile Bootstrap method were used to examine the mediating effect. According to the mediation effect analysis program proposed by Zhao [39], and the bootstrap method proposed by Preacher and Hayes [40], the mediation effect test was carried out with the sample size of 5000. Under the 95% confidence interval, the result of the mediation test did not contain 0 (except for conscientiousness). The results showed that neuroticism, agreeableness, openness, and extroversion had significant mediating effects, and the mediating effects were − 0.037, -0.063, -0.033, and − 0.040, respectively. In addition, mental workload can directly, and indirectly affect the PPB. Neuroticism, agreeableness, openness, and extroversion can mediate the impact of mental workload on the PPB, but they are not completely mediating, they are partially mediated, as shown in Fig. 2, and Table 4.

**Table 4:** Analysis of the influence of mental workload, and personality on perceived professional benefits of nurses

Mediating effect		Path	Effect	SE	T value	P value	Proportion of mediating effect
Mediating effect 1	Total effect						0.103
		MW-PPB	-0.355	0.056	-6.315	0.000	
	Direct effect						
		MW-PPB	-0.319	0.058	-5.516	0.000	
	Indirect effect			Boot SE	BootLLCI	BootULCI	
		MW-N-PPB	-0.037	0.019	-0.081	-0.007	
Mediating effect 2	Total effect						/
		MW-PPB	-0.355	0.056	-6.315	0.000	
	Direct effect						
		MW-PPB	-0.353	0.057	-6.234	0.000	
	Indirect effect			Boot SE	BootLLCI	BootULCI	
		MW-C-PPB	-0.003	0.006	-0.020	0.007	
Mediating effect 3	Total effect						0.178
		MW-PPB	-0.355	0.056	-6.315	0.000	
	Direct effect						
		MW-PPB	-0.292	0.060	-4.884	0.000	
	Indirect effect			Boot SE	BootLLCI	BootULCI	
		MW-A-PPB	-0.063	0.021	-0.110	-0.067	
Mediating effect 4	Total effect						0.093
		MW-PPB	-0.355	0.056	-6.315	0.000	
	Direct effect						
		MW-PPB	-0.322	0.058	-5.536	0.000	
	Indirect effect			Boot SE	BootLLCI	BootULCI	
		MW-O-PPB	-0.033	0.015	-0.066	-0.007	
Mediating effect 5	Total effect						0.114
		MW-PPB	-0.355	0.056	-6.315	0.000	
	Direct effect						
		MW-PPB	-0.315	0.058	-5.426	0.000	
	Indirect effect			Boot SE	BootLLCI	BootULCI	
		MW-E-PPB	-0.040	0.016	-0.072	-0.014	

Note: MV = mental workload; PPB = perceived professional benefits; N = Neuroticism; A-Agreeableness; E = Extraversion; O = Openness; C = Conscientiousness

## Discussion

This study investigated the status quo of mental workload, personality, and PPB of nurses in tertiary hospitals in East China, as well as the influence of social-demographic factors on PPB, analyzed the mediating role of personality(neuroticism, conscientiousness, agreeableness, openness, and extroversion) in mental workload, and PPB, and aimed to explore the specific mechanism of personality(neuroticism, conscientiousness, agreeableness, openness, and extroversion) in the effect of mental workload on PPB from an individual perspective.

The score of the PPB in this study was 128.00, which was above the median value(33*3 = 99), but lower than the research result of Liu [41], we speculated that the main reason is that the subjects were different, Liu’s research objects were nurses who were supporting Wuhan’s anti-epidemic work. The national health department, and the public had significantly improved the cognitive values of nurses, and their conditions of personal protective equipment, remuneration, and other conditions had also improved markedly. There were other professional benefits, such as economic returns, social support, confidence, and certificates of honor [41]. The PPB of nurses in this study was lower than that of ICU nurses [42]. Considering that ICU workers are trained to deal with emergency changes, and critical conditions, the PPB can also be improved. Managers should take measures to promote the connection between nurses, and patients, perfect the professional image of nurses, and strengthen the positive occupational perception of nurses, to improve the PPB of nurses.

The mental workload of nurses in this study was higher than the median value (according to the scoring method of the scale, the total score is the arithmetic mean of all the item score. 6*50/6 = 50). It was also higher than Pourteimour’s study of Iranian ICU nurses [43], and the scores of each dimension were slightly lower than the study result of Liang [34]. Considering the different development speeds in Hangzhou, Shanghai, Iran’s Urmia, and Hamadan, the different pressures in the medical service industry was caused by the different economic pressures, the better the economic status, and development level of a region, the greater the use of health services by people. People will consider their own health status, and use medical services according to needs [44]. However, the increasing demands for medical, and nursing services are in contradiction with the limited nurse-patient ratio, which directly leads to the increase of nurses’ workload. So, it suggested that nursing managers should guide nurses to recognize, and react workload positively.

In this study, the scores of conscientiousness, and agreeableness were the highest, while the score of neuroticisms was the lowest. Similar to the research results of Huang, and Lin [45, 46], the high score of conscientiousness indicates that nurses have higher self-restraint ability, a sense of responsibility, are orderly, and are rule-followers [47]. A high agreeableness score indicates that nurses are helpful, reliable, compassionate, cooperative, and modest [48]. In addition, Studies have found that individuals with high neuroticism are emotionally unstable, and have many psychological problems, such as anxiety, depression, hostility, and impulsiveness [49, 50]. Negative or neurotic perfectionism often has negative characteristics, such as neurotic disorders, negative emotions, excessive feelings of pressure, ineffective coping strategies, and low subjective well-being [51]. Emotionally centered coping strategies often adopted by neurotic individuals are closely related to lower life meaning, and job satisfaction [52]. In this study, nurses scored the lowest in this dimension(Neuroticism), indicating that nurses are emotionally stable, have a good interpersonal relationships, and care for others, which is conducive to building a good nurse-patient relationship.

This study found that the nurses aged 25–35 years old had higher PPB than those nurses aged > 40 years old, which was different from Meng’s [53] study result that age did not affect nurses’ perceived professional benefits. And the nurses with less than 2 years of work service, and 6–10 years of work service had much more PPB than those of > 20 years work service, However, it is different from Zhan’s [54] research, which believed that the nurses aged 40 years and above, and those with higher professional titles have higher perceived professional benefits. Considering that the subjects of this study were mostly 20–40 years old, these nurses with elder age or longer length of service in nursing may lead to job burnout, while those with less than 2 years of work service were just beginning to engage in nursing. Junior nurses felt the benefits of stable nursing job, high-quality medical resources, health care, and the ability to take care of themselves, so their professional experience was positive [55]. Nurses with 6–10 years of work service are in the stage of high intelligence, physical strength, and efficiency, are passionate about their work, and have more career development opportunities, so their professional benefit is the highest.

This study also found that the PPB of nurses with technical secondary school, and undergraduates’ degree were higher than those with specialized subject degree, which was different from Zhao’s [56] research outcome that nurses with lower education degree perceived more benefits in positive professional perception, and Wang [57] thought that educational background would not affect the formation of PPB. The main consideration is that China’s nurses whose educational background is technical secondary school are often 40–50 years old, with low educational background but relatively good economic, and social status in accordance with the times trend, so their PPB is high. However, there is an employment discrimination against specialist nurses in Class A tertiary hospitals at present, their PPBs are low compared with those bachelor degree’s nurses.

This study found that gender, labor and personnel relations, working department, professional title, and marital status did not affect nurses’ PPBs. Consistent with the results of Wang [58], and Guo’s [59] research result that gender, and department do not affect PPB, and different from Zhao’s [56] research that the level of PPB of nurses whose labor and personnel relations is having establishment is higher than that of without establishment nurses, considering that the current level of equal pay for equal work is gradually realized in the personnel management of Hangzhou Class A tertiary hospitals, and the gap in welfare benefits is getting smaller.

This study found that mental workload was negatively correlated with the PPB, H1 hypothesis is confirmed, indicating that excessive mental workload would lead to emotional exhaustion, work neglect, workplace rejection, and difficulty in devoting oneself to work. The high workload, and high-pressure working environment will have a certain negative impact on the physical, and mental health of nurses [60], about 18% of nurses were forced to resign due to heavy work [61]. This study also found that mental workload is positively correlated with neuroticism, and negatively correlated with agreeableness, openness, extroversion, and conscientiousness, H2 hypothesis is confirmed. The findings are similar to a Danish study [62]. Considering that individuals with agreeableness, openness, and extroversion are good at communication, have good social support, and are more likely to deal with pressure, neurotic individuals lack emotional stability, are self-centered, have poor social relations, and may overestimate their feelings of pressure [63]. Studies have also found that individuals with high conscientiousness, agreeableness, extraversion, low neuroticism, and low psychoticism will cope with pressure actively [64], and obtain high social support, so they have a strong ability to bear pressure. The more extroverted a person is, the healthier they are, and the higher their job satisfaction, whereas neuroticism tends to be negatively correlated with these results [65, 66].

This study found that neuroticism, and PPB negatively correlated, conscientiousness, agreeableness, openness, and extroversion were all positively correlated with PPB, H3 hypothesis is confirmed. Personality is the sum of psychological characteristics with a certain tendency, representing a person’s overall mental outlook. People with positive, and optimistic personality have good social adaptability [67]. Nurses with neurotic personality tendencies are prone to tension, anxiety, irritability, and other psychological states in clinical work, and they are also sensitive, suspicious, and hostile [68]. They are prone to suffer negative responses when dealing with problems, and interpersonal relationships, thus wasting their PPB. Other studies have also reached similar conclusions. For example, the sense of responsibility, openness, and extroversion in personality can significantly positively predict college students’ life meaning; Neuroticism, on the other hand, significantly negatively predicted life meaning, and job satisfaction [69, 70]. Conscientiousness, and openness to experience in team personality have a positive effect on team organizational fitness, and teamwork fitness, to promote teamwork performance, and job satisfaction [71]. Conscientiousness, and agreeableness partially mediated the relationship between family dynamics, and sleep quality [72]. This study found that neuroticism negatively predicted PPB, while agreeableness, and extraversion positively predicted PPB, but the difference was not significant, which was similar to Chang’s study [70]. It is also similar to Huang’s study that high scores of introversion, neuroticism, and psychoticism, and strong workload are risk factors for individual nervous response, physical, and mental health [73], indicating that nursing work needs to be empathetic, empathic, caring about patients’ agreeableness, and extroversion that is good at communication, and expression.

At the same time, it is found that personality (neuroticism, agreeableness, openness, and extroversion) played a mediating role in mental workload, and PPB, the indirect mediating effects of agreeableness, extraversion, neuroticism and openness were 17.8%, 11.4%, 10.3% and 9.3%, respectively, the H4 hypothesis proved to be true. This is similar to the conclusion of Zhan [28] that personality of Guangdong Railway police played a mediating role in job stress, and happiness, also similar to the results that compassion satisfaction, burnout played a chain mediating role in the relationship between extraversion, agreeableness, conscientiousness, openness, and job satisfaction (the positive effects), and played a negative role in mediating neuroticism, and job satisfaction [74]. This study also found that conscientiousness was related to PPB, but the mediating effect was not obvious. Considering conscientiousness is only a passive response to the pressure in work, rather than the subjective pleasure or sense of gain arising from the response to the pressure. Some studies suggested that conscientiousness has a direct positive impact on problem-solving strategies, but the degree of positive impact on cognitive restructuring is small [75]. It is worth nursing managers’ attention that nurses with different personalities have great differences in their feelings under the same pressure, then have different ways of dealing with them, resulting in various emotional responses [76], so there are differences in their job engagement, burnout, and perceived professional benefits. A study found that the strongest tendency of nurses is agreeableness [77]. Nurses with this trait have such characteristics as caring, and altruism, are more likely to be treated well by others, and get positive feedback from good interpersonal relationships in the work environment, thus having higher job satisfaction [78]. The higher the extroversion tendency indicates that nurses have rich imagination, and feelings, are easier to perceive others’ emotions in clinical practice, have empathy [79], and are conducive to social interaction. Openness refers to a tendency toward aesthetics, various experiences, and creativity [80]. These personalities above mentioned all show the willingness of individuals to strengthen communication with others, better social adaptation, and innovation ability, which proves that good social relationships can help improve happiness. Therefore, it is suggested that nursing managers should focus on guiding the cultivation of nurses’ communication ability, empathy, and innovation ability, to better meet the requirements of nurses’ professional role characteristics. Mindfulness and compassion training programs develop patient-centered care relationships [81]. Role-playing training [82], Virtual Counseling Application [83], improving nurses’ self-efficacy [84] may be good methods to promote communication ability. Virtual patient simulation to improve nurses’ relational skills either [85]. In terms of improving nurses’ empathy, social-cognitive mindfulness emphasizes strong re-evaluation [86], Loving-kindness meditation [87], narrative medicine theory education based on online platform, and narrative writing contribute to the development of empathy, communication, and humanistic care ability [88]. Information and Communication Technology can facilitate disruptive innovation among emergency department nurses [89]. Innovation workshops can enhance nurses’ creativity [90]. Another priority is to strengthen the emotional counseling, and stress coping of neurotic nurses, such as teaching them relaxation training, guided reflexes, breathing training, active meditation training, aromatherapy, music therapy, etc., to help them regulate their emotions [91]. In addition to this, according to the JD-R model, the mental workload can be considered as a kind of job demand, while nurses’ PPB are a job resource. To reduce mental workload, the following measures can be taken to improve nurses’ PPB according different personalities. First, it should reduce the nursing workload, increase the number of nurses, increase labor remuneration, and give appropriate shifts so that the nurses can recuperate from illness, and strengthen capacity to cope with workload, which all can minimize the perceived stress level of nurses [92]. At the same time, it is necessary to rearrange, and allocate nursing staff according to the difficulties of the nursing work when necessary [93]. Secondly, it is worthy of attention that managers should attach importance to the personality, and occupation matching of nursing professionals. Holland personality test should be used at the beginning of new nurses’ employment, and it found that the nurses with obvious neuroticism tended to avoid the work in contact with patients as much as possible, and arrange appropriate workload according to individual ability. Thirdly, managers should adopt strategies, such as emotional support, and professional training to enhance nurses’ positive perceptions, and improve their attitude toward their profession. Cognitive intervention on perceived professional benefits [94], Mindfulness-based stress reduction training [95] can help nurses improve their PPBs, relieve their professional slack, promote their physical, and mental health, and improve the nursing quality. Making good use of the positive effects of challenging stress [96], Satir model training [97] all can improve nurses’ PPBs. Fourthly, improving the working environment proved to be an effective way to reduce mental workload, and improve the PPBs. The nurse’s professional environment could play an intermediary role between perceived occupational support, and nurses’ PPB [10]. Therefore, it is necessary to form an atmosphere of understanding the hard work of nurses, and respecting the efforts of nurses from the national health system, and the social public aspect, improve the working remuneration of nurses, develop a friendly, and harmonious working environment in hospitals, and departments, train nurses’ clinical communication skills, and reduce the conflicts between nurses, and patients, all of which are conducive to improving nurses’ PPBs. Fifthly, it should create more learning opportunities to improve nurses’ abilities to enhance nurses’ PPBs. Finally, long-term assessment of nurses’ perception of workload, and regular training of positive psychological resources (self-efficacy [10], resilience [41]) are necessary to improve nurses’ PPBs, and promote their mental health.

### Limitations

This study provided a certain empirical basis for nurses with different personalities to cope with the workload, and improve their PPB, discussed the mediating role of personality in the mental workload on PPB, and established the connection between job demands, and job resources. It is worth noting that this study only takes parts of nurses in Class A tertiary hospitals in Hangzhou as the subjects. Therefore, the conclusions on the influencing factors of nurses’ PPB may be one-sided, lacking sampling of nurses from other hospitals (Such as primary, and secondary hospitals). Therefore, the sample source is derived from a capital city of an economically developed province in East China, which cannot represent the situation of the whole country. The methods utilized in this study were self-assessment questionnaires, which lacked objective data. If the findings on the relationship between mental workload, personality, and PPB need to be carefully extrapolated to other regions. Future research should be conduct to explore the other influencing factors on PPB of hospital nurses more thoroughly, and it is essential to actively explore the empirical effects of intervention.

## Conclusion

The results of this study have positive significance for nursing management practice. It is very important to improve the perceived professional benefits of nurses with different personalities according to their perception of mental workload, to improve the nursing quality, and reduce the turnover rate of them. Our research results showed that personality has a certain mediating effect between mental workload (job demand), and perceived professional benefits (job resources). In order to make the personality, and ability of nurses more match with the characteristics of nurses’ professional roles, specific strategies should be adopted, such as guiding nurses with different personalities to face negative emotions, and mental workload positively. Training should focus on improving nurses’ communication, empathy, and innovation abilities.

## Data Availability

The identified datasets analyzed during the current study are available from the corresponding author on reasonable request.
